# Editorial: World breastfeeding week 2024: an obstetric and pediatric pharmacology perspective

**DOI:** 10.3389/fphar.2025.1671327

**Published:** 2025-08-05

**Authors:** Olusola Olafuyi, Amita Pansari, Philip O. Anderson, Karel Allegaert

**Affiliations:** ^1^ Division of Physiology, Pharmacology and Neuroscience, School of Life Sciences, University of Nottingham, Nottingham, United Kingdom; ^2^ Predictive Technologies Division, Certara UK Limited, Sheffield, United Kingdom; ^3^ Skaggs School of Pharmacy and Pharmaceutical Sciences, University of California San Diego, La Jolla, CA, United States; ^4^ Department of Development and Regeneration, KU Leuven, Leuven, Belgium; ^5^ Department of Pharmaceutical and Pharmacological Sciences, KU Leuven, Leuven, Belgium; ^6^ Department of Hospital Pharmacy, Erasmus MC, Rotterdam, Netherlands

**Keywords:** breastfeeding, lactation, clinical pharmacology, lactation related drug exposure, safety, modelling and simulation, regulatory

## Introduction

World breastfeeding Week (WBW) is celebrated annually in the first week of August, organized by the World Alliance for Breastfeeding Action (WABA) the World Health Organization (WHO), and United Nations International Children’s Emergency Fund (UNICEF), aiming to promote breastfeeding and its many benefits to mother and child (World Alliance for Breastfeeding Action, 2025; World Health Organization, 2025a). WHO recommends exclusive breastfeeding for the first 6 months of life, and if possible, continuing with supplemented breastfeeding for a further 1–2 years. These initiatives have significantly improved breastfeeding practices (World Alliance for Breastfeeding Action, 2025; World Health Organization, 2025a). Over the last 3 decades, death rates and disability-adjusted life years due to suboptimal breastfeeding reduced by approximately 80%, reflecting its relevant health benefit (Zhu et al., 2025). Unfortunately, while the rates are increasing, WHO recommendations such as exclusive breastfeeding in the first 6 months are still only very partially attained. To raise the exclusive breastfeeding rate towards the WHO goals (achieving 70% exclusive breastfeeding at 6 months by 2030), targeted interventions are needed whereby participation of each stakeholder is crucial (World Alliance for Breastfeeding Action, 2025; World Health Organization, 2025b).

Achieving this goal will require a better understanding of regional socio-economic drivers to develop tailored interventions, as well as the shared obligation to generate knowledge on pharmacotherapy and its safety during breastfeeding (World Health Organization, 2025b; Van Neste et al., 2025). There is still a huge information gap (“information desert”) on medication safety during breastfeeding; even though most women require at least one medicine postpartum, in part due to a rising number in chronic diseases and pregnancies at later age (Anderson, 2018; Fomina et al., 2023).

The goal of this Research Topic is to bring to the forefront critical research on breastfeeding from an obstetric and pediatric perspective, exploring a range of research strategies, involving *in vitro*, *in vivo* or computational techniques to address drug-related challenges associated with breastfeeding. We intended to present diverse perspectives and encouraging interdisciplinary collaboration to deepen understanding of the implications of drug use during lactation on both mother and child. The Research Topic has 7 papers, offering a diverse range of perspectives, either focusing on how to improve computational techniques to improve modeling and simulation in this specific population (Heo et al.; Deferm et al.), on drug-related problems in lactating women (Morze et al.; Morze et al.), or describing drug-specific (Den Besten-Bertholee et al.; Van Neste et al.) or more generic approaches to assess safety during lactation-related drug exposure in the infant (Monfort et al.).

## Modeling and simulation advances

Related to improvement of computational techniques, Heo et al. explored the performance of mathematical models to predict medicine distribution into breastmilk, and concluded that the biological heterogeneity of breastmilk has a major impact on model development and performance. The authors therefore call for improved understanding of the physiological mechanisms to improve *in silico* methodologies (Heo et al.). Strongly related to this call, Deferm et al. reported on the development of a comprehensive database on postpartum changes in maternal physiology and milk composition to support these *in silico* methodologies (Deferm et al.). Based on a dataset with 36,689 data points from 20,801 postpartum women (childbirth, up to 1 year afterwards), mathematical functions were generated for a diversity of physiological parameters, capturing postpartum changes over time.

## Drug-related problems in clinical practice

On drug-related problems, two papers from the Poznan group are provided. A first paper, has a clear call “to mind the gap” among breastfeeding women. In a prospective study in 200 breastfeeding women who registered for a pharmacist’s online consultation, 190/200 reported on any drug-related problems (DRPs), and 163/200 manifested actual DRPs, including ineffective therapy (138/163), untreated symptoms or indications (16/163), or potential adverse drug reactions (9/163). The most common causes (Naranjo, Liverpool Causality Assessment Tool) were patient-related factors, or dispending-related Research Topic (Morze et al.). In a subsequent study with focus on DRPs in 47 breastfeeding women treated for depressive spectrum disorders, 49 DRPs were identified, with inadequate treatment effect due to underdosing or not taking the drug being the most common (57%), followed by possible adverse event (22%), untreated symptoms or indications (14%), or no effect despite correct use (6%) (Morze et al.). Considering the limitations like sample size, biases and limitations related to cohort construct and healthcare setting, the approach described can be applied to better capture DRPs in this setting.

## Drug-specific and generic approaches to assess safety

Finally, case series on sertraline, citalopram and paroxetine, or clopidogrel quantified the passage of these specific medicines into breastmilk and the subsequent infant exposure (Den Besten-Bertholee et al.; Van Neste et al.). The authors concluded that - given the well-known benefit of breastfeeding (World Alliance for Breastfeeding Action, 2025; World Health Organization, 2025a) - their findings support breastfeeding of infants by mothers who are taking these medicines.

In addition to these medicine specific observations, and somewhat merging the Research Topic on computational techniques with case series reporting, Monfort et al. reported on a “Milk4baby decision tree approach”, describing a workflow for pragmatic and contextualized method selection on how to assess safety of infant systemic medicine exposure through human milk in clinical trials or care (Monfort et al.). To address this, the authors identified key medication-related characteristics essential for designing lactation studies that assess infant safety following systemic exposure during lactation: the prevalence of a given medication utilization in women of childbearing age, the medication’s safety profile in infants (0–2 years), and the expected infants’ systemic exposure.

## How this Research Topic support the regulatory efforts

The final concept paper E21 of the International Conference of Harmonisation clearly calls for earlier incorporation of clinical data in product labelling to inform decisions (International Conference of Harmonization, 2023). While the global increased interest in this area is encouraging, there is a risk that development of multiple guidelines could introduce complexity due to inconsistent recommendations, so that harmonization is warranted (International Conference of Harmonization, 2023). Specific considerations to ensure safe data acquisition and study design are the use of existing data sources, real world evidence of use and needs in breastfeeding (cf DRP), and the availability of physiologically based models.

## Discussion and conclusion

This Research Topic has limitations on its diversity of Research Topic since e.g., bio-analysis, data analysis incorporating maternal medical conditions, or ethics are not present. However, based on the newly reported information on how to improve computational techniques, on DRPs in lactating women, and on drug-specific or generic approaches to assess safety of drug exposure during lactation contributes to this overarching aim to convert the ‘information desert’ into a more sustainable environment for all stakeholders involved.
